# Adaptive Potential of Epigenetic Switching During Adaptation to Fluctuating Environments

**DOI:** 10.1093/gbe/evac065

**Published:** 2022-05-09

**Authors:** Dragan Stajic, Claudia Bank, Isabel Gordo

**Affiliations:** Instituto Gulbenkian de Ciência, 2780-156 Oeiras, Portugal; Department of Zoology, University of Stockholm, Stockholm, Sweden; Department of Infectious Diseases and Pathobiology, Institute for Fish and Wildlife Health, University of Bern, 3012 Bern, Switzerland; Instituto Gulbenkian de Ciência, 2780-156 Oeiras, Portugal; Department of Biology, Institute for Ecology and Evolution, University of Bern, 3012 Bern, Switzerland; Swiss Institute of Bioinformatics, 1015 Lausanne, Switzerland; Instituto Gulbenkian de Ciência, 2780-156 Oeiras, Portugal

**Keywords:** epigenetic switching, mutations, adaptation, fluctuating environments

## Abstract

Epigenetic regulation of gene expression allows for the emergence of distinct phenotypic states within the clonal population. Due to the instability of epigenetic inheritance, these phenotypes can intergenerationally switch between states in a stochastic manner. Theoretical studies of evolutionary dynamics predict that the phenotypic heterogeneity enabled by this rapid epigenetic switching between gene expression states would be favored under fluctuating environmental conditions, whereas genetic mutations, as a form of stable inheritance system, would be favored under a stable environment. To test this prediction, we engineered switcher and non-switcher yeast strains, in which the uracil biosynthesis gene *URA3* is either continually expressed or switched on and off at two different rates (slow and fast switchers). Competitions between clones with an epigenetically controlled *URA3* and clones without switching ability (*SIR3* knockout) show that the switchers are favored in fluctuating environments. This occurs in conditions where the environments fluctuate at similar rates to the rate of switching. However, in stable environments, but also in environments with fluctuation frequency higher than the rate of switching, we observed that genetic changes dominated. Remarkably, epigenetic clones with a high, but not with a low, rate of switching can coexist with non-switchers even in a constant environment. Our study offers an experimental proof of concept that helps defining conditions of environmental fluctuation under which epigenetic switching provides an advantage.

**Significance**

Through the epigenetic regulation of gene expression, a single genotype can produce several phenotypes, establishing phenotypic heterogeneity in an, otherwise, genetically uniform population. However, these epigenetically determined phenotypes are not as stable as phenotypes determined by genetic changes, and they can frequently switch between the alternative forms. Due to its high level of instability, the role of epigenetic mechanisms in evolutionary processes is still contentious. Here, we tested under which environmental conditions epigenetic switching could be favored. We show that even though genetic changes dominate adaptation across environments, epigenetic inheritance seems to strive under particular fluctuating environments and is maintained at low frequency even under conditions that were predicted to be unfavorable for epigenetic switchers.

## Introduction

Natural populations experience variable, often fluctuating, environmental conditions that can exert significant selective pressures. Survival of populations under such changing conditions depends, in part, on the level of phenotypic heterogeneity within the population (Lande 1976; Bruijning et al. 2020). Such phenotypic diversity can be established through the maintenance of genetic diversity or through mechanisms by which the same genotype can produce alternative phenotypes (Cohen 1966; Seger and Brockmann 1987; Bruijning et al. 2020). Such phenotypes might be maladapted in the current environment, but could potentially provide a net fitness benefit upon the environmental change (Seger and Brockmann 1987). In other words, the mechanisms of maintenance of phenotypic heterogeneity might minimize the arithmetic mean fitness of the population, but maximize the geometric mean fitness across environments (Gillespie 1977; Seger and Brockmann 1987; Bruijning et al. 2020). This represents the conceptual core of a bet-hedging survival strategy (Slatkin 1974).

Phenotypic heterogeneity in an isogenic population can be established through self-reinforcing feedback loops that maintain distinct gene expression states independently of the underlying sequence (Bird 2007; Jablonka and Raz 2009; Moazed 2011). These epigenetically determined phenotypic states are typically less stable than those caused by genetic changes and can intergenerationally switch stochastically between different gene expression states. The rate of epigenetic switching between distinct phenotypes was shown to be several orders of magnitude higher than the mutation rate (Dodson and Rine 2015; Van Der Graaf et al. 2015). Due to the high rate of instability of epigenetic states, the potential contribution of epigenetic switchers to the evolutionary processes is contentious (Charlesworth et al. 2017).

Theoretical work has shown that phenotypic heterogeneity and stochastic switching between the phenotypic states are favored during adaptation in fluctuating environments, where each of different reiterated environmental conditions select for a specific distinct phenotype (Lachmann and Jablonka 1996; Thattai and Van Oudenaarden 2004; Kussell and Leibler, 2005; Rajon and Charlat 2019). Here, epigenetically induced phenotypic heterogeneity could provide the basis for a bet-hedging survival strategy (Cohen 1966; Beaumont et al. 2009).

The advantage of a phenotypic switching mechanism depends on the rate of environmental fluctuations (Kussell and Leibler 2005). The optimal switching rate is expected to be the one that matches the average frequency of environmental fluctuations, that is, 1/T, with T being the time interval of environmental change (Lachmann and Jablonka 1996). As the environmental period becomes shorter, the optimal phenotypic switching rate increases. Under such conditions, genetic mutations between the two phenotypic states are expected to be of little effect due to their low probability of occurrence compared with the frequency of environmental oscillations (e.g., the genetic mutation rate in budding yeast tends to be several orders of magnitude lower than the epigenetic switching rate [Lang and Murray 2008; Dodson and Rine 2015]).

Recent experimental studies have shown that epigenetic switching can affect the growth rate in fluctuating environments (Acar et al. 2008; Kronholm and Ketola 2018; Proulx et al. 2019). Here, populations with a high rate of epigenetic switching had a higher growth rate upon an environmental change compared with populations with a lower rate of switching (Acar et al. 2008).

Theoretical and empirical studies have shown that, on the other hand, in a constant environment, where conditions are unchanging during the course of adaptation, genetic mutation is the favored form of adaptation (Rajon and Charlat 2019). In a constant environment, epigenetic switching can provide an initial advantage and promote the fixation of beneficial genetic mutations (Lachmann and Jablonka 1996; Paenke et al. 2007; Bódi et al. 2017; Stajic et al. 2019; Torres-Garcia et al. 2020).

Additionally, epigenetic switching can affect the nature of mutational targets and their fitness effects (Charlebois 2015). Epigenetic gene expression states could effectively increase cryptic genetic diversity within a population by reducing the selective constraint on genetic determinants. For example, epigenetic silencing of a gene could render all subsequent mutations in that gene neutral (Klironomos et al. 2013). Here, an epigenetic system of inheritance might allow populations to explore the fitness landscape and facilitate the transition between fitness peaks (Pál and Miklós 1999, Tadrowski et al. 2018). Furthermore, epigenetic systems were also shown to change the spectrum of beneficial mutations during adaptation, enabling acquisition of adaptive mutations that modulate epigenetic control of gene expression (Stajic et al. 2019).

The advantage of an epigenetic switching system will depend on the rate of switching and the fitness of the phenotypes it produces (Tadrowski et al. 2018). Certain rates of switching seem to be maladaptive and hinder long-term adaptation (Kronholm and Collins 2015).

However, the direct measurement of the advantage of an epigenetic system of inheritance compared with genetic mutations, under different environmental conditions, has been difficult to ascertain. We designed an experimental setup in which theoretical predictions can be directly evaluated by competing a yeast strain containing an epigenetic machinery (with different rates of epigenetic switching) with a strain that can only adapt through genetic mutation (created via a knockout of epigenetic silencing components). We ask two main questions: (1) Under periodic environmental changes, can epigenetically induced phenotypic stochasticity become dominant in the populations? (2) Under different environmental conditions, fluctuating or stable, which inheritance system would be more important?

We used previously constructed and well-characterized *Saccharomyces cerevisiae* strains, in which a *URA3* reporter gene was inserted into a subtelomeric region (Stajic et al. 2019), resulting in differential epigenetic silencing of the gene. *URA3* is a widely used reporter gene that enables dual selection (i.e., selection for gene activation and inactivation). The gene is crucial for the production of uracil, which is essential for cell growth. However, in the presence of a drug, 5-fluoroorotic acid (5-FOA), the activity of the Ura3 protein is deleterious, because it converts the drug into a toxic 5-fluorouracil that kills the cell (Boeke et al. 1987). The strength of the silencing and the rate of switching between ON and OFF state of *URA3* expression in the subtelomeric region are dependent on the activity of silent information regulator (SIR) proteins (Ivy et al. 1986; Rine and Herskowitz 1987; Aparicio et al. 1991), which act as chromatin modifiers (Imai et al. 2000), and the relative distance of the gene from the telomere (Pryde and Louis 1999). Using this well-established system, we selected *URA3* gene activation (ON state) by removing uracil from the medium, or inactivation (OFF state) by adding 5-FOA.

Our study provides an experimental proof of concept that quantifies the dynamics of epigenetic switching during adaptation to environments with different periodicity of fluctuations. We observe that genetic mutations are the predominant mechanism of adaptation across different rates of environmental fluctuations. However, we find that under specific environmental conditions, an epigenetic form of inheritance is favored over genetic mutations. Surprisingly, we find that clones capable of epigenetic switching can coexist with non-switchers even in stable environmental conditions, where genetic mutations are expected to provide a higher adaptive advantage.

## Results

### Mutations are Favored Over Epigenetic System of Inheritance in Stable Environments

**Fig. 1.**
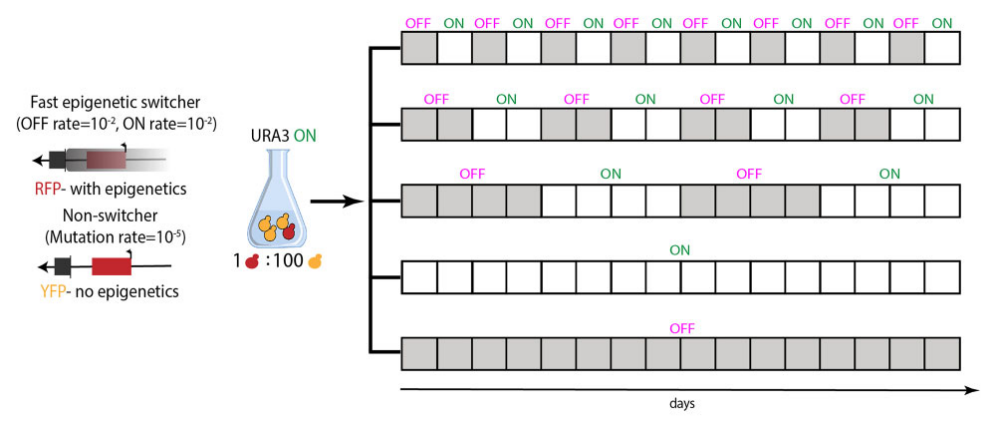
Experimental setup. Scheme showing experimental evolution setup used in the study. Yeast strains with epigenetic silencing (labelled with RFP) and a *SIR3* knockout strain (labelled with YFP) were preselected in media without uracil (i.e., selection for active *URA3* gene) and mixed in 1:100 proportions, respectively. Subsequently, the mixed RFP/YFP yeast cultures were exposed to environments that fluctuated with different periodicity. Each box represents a 24-h period after which populations were sampled and relative ratio of strains were determined. The color of the boxes represents the selection regime whereby gray boxes indicate selection for the inactive form of *URA3* and white-colored boxes indicate selection for the active form of *URA3*.

We chose a strain, referred to as fast epigenetic switcher, with subtelomeric *URA3* position that showed high levels of epigenetic switching between ON and OFF gene expression state: ON rate  ≈ 10^−2^, OFF rate  ≈ 10^−2^ (Stajic et al. 2019). We directly competed this strain with its corresponding Δsir3 mutant (referred to as non-switcher) that lacks an essential component of the SIR machinery and, consequently, lost its ability to epigenetically control gene expression in the subtelomeric region. Moreover, the non-switcher strain was previously shown to have a mutation rate of 10^−5^ (Stajic et al. 2019). The initial population size of 10^6^ cells ensures fast acquisition of beneficial mutations in this background. This system allowed us to monitor, in a controlled manner, the effect of epigenetic changes and mutations during adaptation by determining the relative frequency of each strain in the population. To distinguish the two strains, each was marked with a different fluorescent marker; the epigenetic switcher with red fluorescent protein (RFP) and the non-switcher strain with yellow fluorescent protein (YFP). In all the competition experiments, we preselected the cells of both switcher and non-switcher strains to be in ON state at the onset of the experiment, by growing the cultures in complete synthetic media (CSM) lacking uracil. We mixed the epigenetic switcher strain with the non-switcher in a proportion of 1:100 to minimize the probability of mutations in the epigenetic switcher background. To test the prediction that an epigenetic switcher should invade in fluctuating environments, we exposed such cocultures to two alternating environments with periods equal or higher than the epigenetic switching rate, each exerting selection pressures for either ON or OFF state of the *URA3* gene (fig. 1). Additionally, we also followed cocultures in the two non-fluctuating environments with constant selection regimes, where we expect populations to be dominated by the non-switcher strain.

**Fig. 2.**
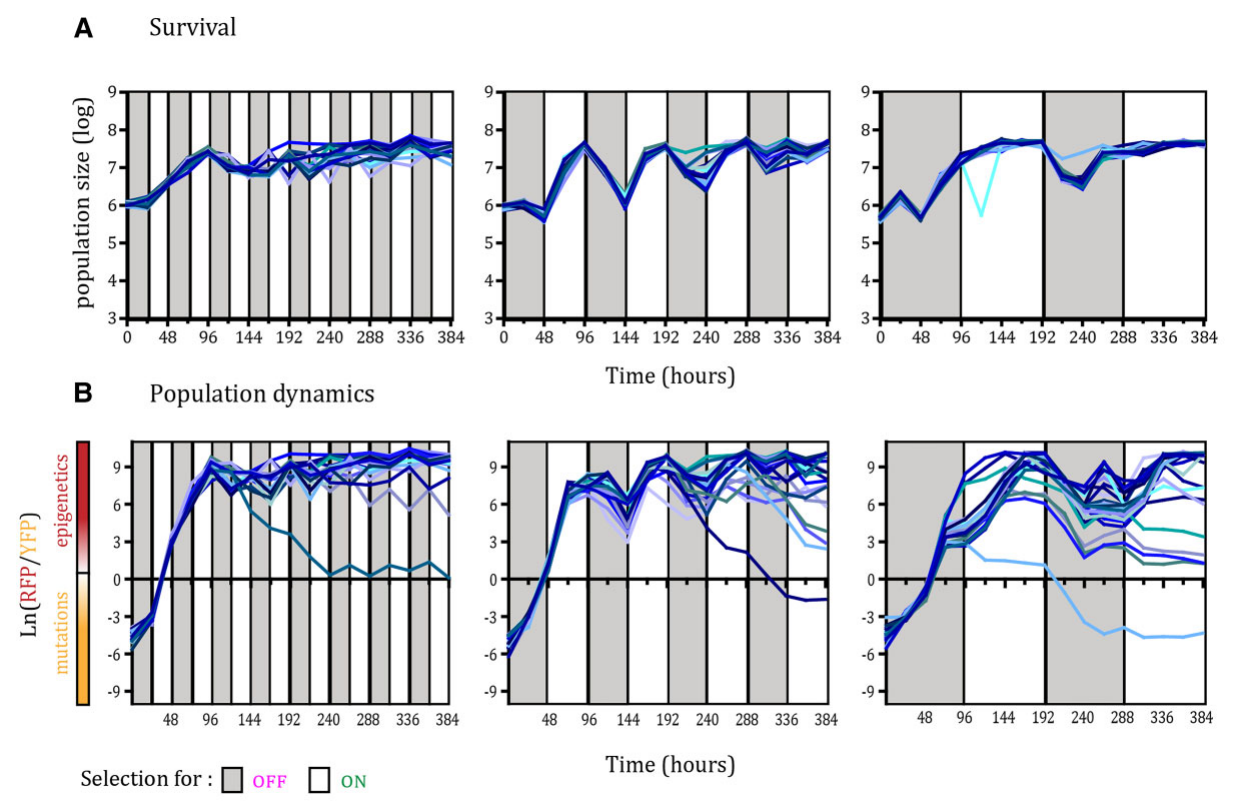
Epigenetic switchers dominate during adaptation to fluctuating environments. (*A*) Survival through the course of selection in the three fluctuating environments with distinct periodicities, determined using FACS methodology. Each line represents the number of cells in each replicate population (24 replicate populations for each environmental condition). Colored areas indicate the selection regime, gray corresponds to selection for inactive *URA3* and white for selection for the active form of the gene. (*B*) Dynamics of RFP/YFP ratios (with high rate of epigenetic switching) in fluctuating environments. The logarithm of RFP/YFP ratios for each of the replicate populations is shown, determined using FACS methodology. The color of the line for each population corresponds to the color of the lines in the survival graphs. Colored areas indicate the selection regime, gray corresponds to selection for inactive *URA3* and white to selection for the active form of the gene. Positive values indicate dominance of the RFP strain, and negative values indicate dominance of the YFP strain.

**Fig. 3.**
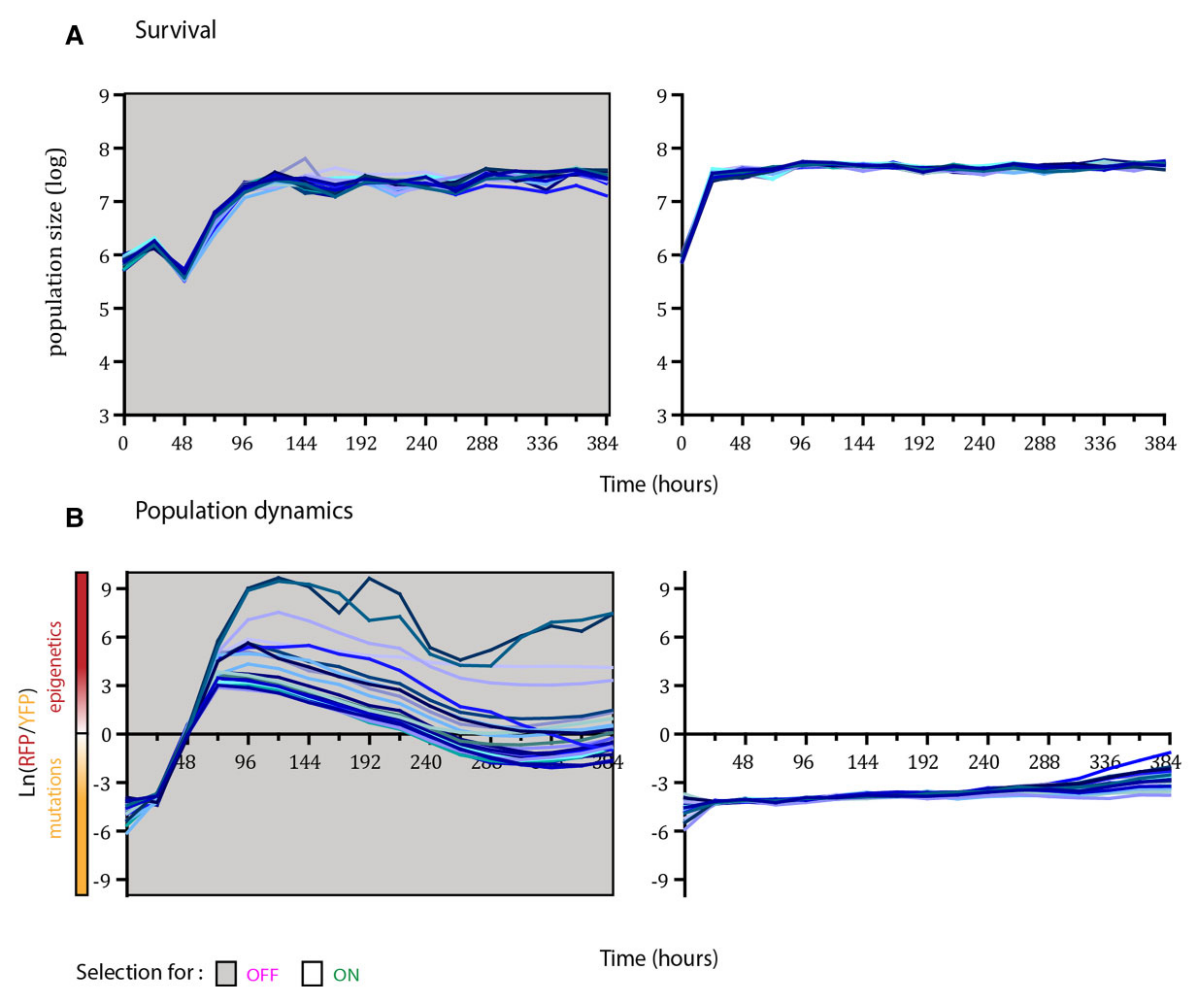
Epigenetic switchers coexist with non-switchers in stable environments. (*A*) Survival through the course of selection for two constant environments with distinct selection regimes here marked with the same colors as in the fluctuating environments (gray indicates selection for an inactive *URA3* gene and white for an active form of the gene), determined using FACS methodology. Each line represents the number of cells in each replicate population over time (24 replicate populations for each environmental condition). (*B*) Dynamics of RFP/YFP ratios in the two constant environments. The logarithm of RFP/YFP ratios for each of the replicate populations is shown, determined using FACS methodology. The color of the line for each population corresponds to the color of the lines in survival graphs. Colored areas indicate the selection regime as in panel A.

**Fig. 4.**
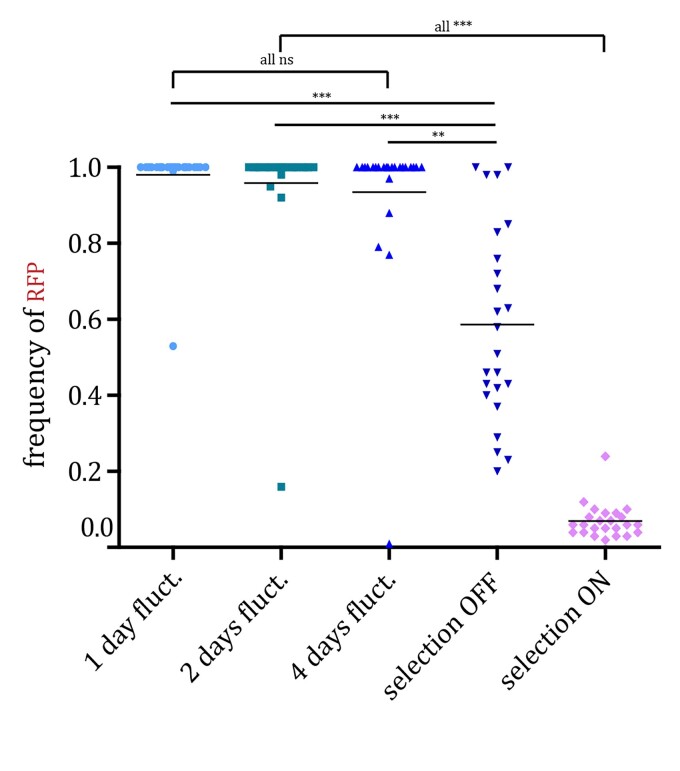
Populations selected in fluctuating environments show a higher frequency of the epigenetic switcher strain than those grown in stable environments. We compared the frequency of the fast epigenetic switcher strain at the final time point across all selection regimes. Points represent frequencies for each replicate population. For each selection regime the mean value across population replicates is plotted. The mean values between the selection regimes were compared using Dunn’s nonparametric test with Bonferroni correction for multiple testing (*n* = 10).

In fluctuating environments, all of the 24 replicate populations of the fast-switching strain survived during the course of the experiment when competing with the non-switcher, irrespective of the periodicity of environmental changes (fig. 2*A*; supplementary table S1, Supplementary Material online). However, in fluctuating environments with 2 and 4 days periodicity, we observed larger oscillations in the total population size than in the fast, 1-day, fluctuating environment, especially during the periods that selected for the OFF state of *URA3* gene expression. Measurement of the relative frequency of the two strains within populations showed that despite the initial predominance of the non-switcher, after 96 h, the majority of cells were epigenetic switchers in all 24 populations of the three fluctuating environments (fig. 2*B*; supplementary table S1, Supplementary Material online). However, after the early sweep of epigenetic switchers through the population, in some replicates the non-switcher recovered by the end of the experiment and increased in frequency above 50% (one replicate in the 2-day fluctuating environment and one replicate in the 4-day fluctuating environment). This indicates that a genetic solution for adapting to these fluctuating environments exists. Nevertheless, at the final time point of the experiment, the frequency of the epigenetic switcher was above 90% in most populations exposed to fluctuating environments (23/24 in 1-day fluctuating environments, 23/24 in 2-day fluctuating environments, and 20/24 in 4-day fluctuating environments). In contrast, under constant selection regimes, only 4/24 populations in 5-FOA environment (selection for OFF state of *URA3*) and 0/24 populations in the environment lacking uracil showed dominance of the epigenetic switcher after 96 h (fig. 3*B*; supplementary table S1, Supplementary Material online). To further quantify this, we compared the mean frequency of epigenetic switchers across all replicate populations for each environmental condition (fig. 4). The mean frequency of epigenetic switchers within populations was significantly higher in fluctuating environments when compared with stable environmental conditions. These results support the theoretical predictions that an epigenetic system of inheritance will have a strong beneficial effect in fluctuating environments, whereas genetic mutations are favored in stable environments.

Even though all of the replicate populations survived in the two constant selection regimes (fig. 3*A*), the population dynamics differed between them (fig. 3*B*). In the stable environment, in which we selected for the OFF state of *URA3* expression, we observed an increase in the relative frequency of epigenetic switchers in the first 72 h (average frequency at 72 h was 98%) due to the high rate of turning *URA3* expression off, which is consistent with the results in fluctuating environments. However, in stable environments, this increase in frequency was followed by the spread of beneficial mutations in the non-switcher background that decreased the relative proportion of epigenetic switchers (average frequency of epigenetic switchers at 384 h was 59%). This indicates that despite the initial benefit of epigenetic switching, in the long-term epigenetically controlled gene expression states are hard to sweep to fixation, probably due to the cost of constantly switching to the less fit phenotype. Nevertheless, the epigenetic switcher persisted in the populations and was maintained at different frequencies until the end of the experiment (∼200 generations; 384 h), resulting in persistent phenotypic and genotypic heterogeneity in the populations.

### The Advantage of Clones with the Epigenetic Machinery Depends on the Period of Environmental Fluctuation

**Fig. 5.**
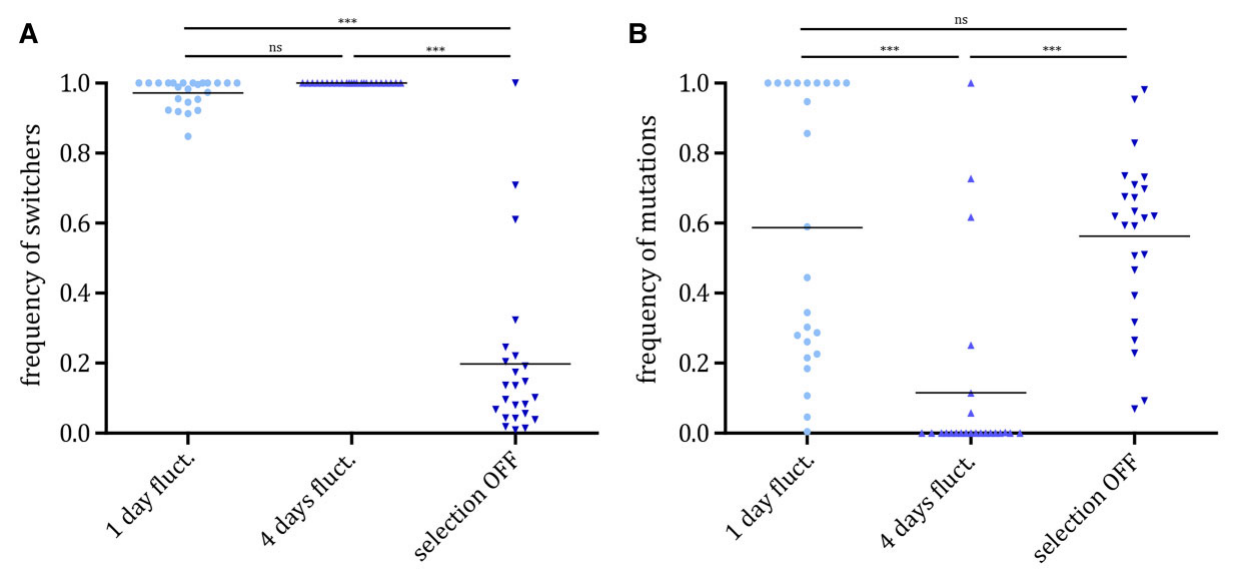
The advantage of epigenetic switching is dependent on the period of environmental fluctuation. (*A*) Points represent frequencies of clones that were able to grow on both the plates containing 5-FOA (selecting for OFF state of *URA3* gene) and the plates lacking uracil (selecting for the ON state of *URA3* gene) within each replicate population at the end of the experiment. Bars represent the mean and standard deviation. The mean values between the selection regimes were compared using Dunn’s nonparametric test with Bonferroni correction for multiple testing (*n* = 3). (*B*) Points represent frequencies of clones whose 5-FOA resistance was not abrogated upon the addition of the inhibitor of epigenetic silencing, nicotinamide, within each replicate population. Bars represent the mean and standard deviation. The mean values between the selection regimes were compared using Dunn’s nonparametric test with Bonferroni correction for multiple testing (*n* = 3).

As mutations can also occur in the switcher background, we set out to confirm that the differences observed in the adaptive dynamics under the different environments are indeed due to epigenetic gene expression regulation. We determined the phenotypes of the evolved clones within each replicate population by plating the replicate populations from the last time point on the nonselective (rich media) plates and subsequently replica plating them onto CSM media plates containing 5-FOA drug and CSM plates that lack uracil. The growth of the evolved clones on both media indicates their ability to epigenetically switch between the active and inactive form of *URA3* gene. On the other hand, the inability of clones to grow on media lacking uracil would indicate a genetic inactivation of the uracil biosynthesis pathway. As our analysis of relative switcher/non-switcher frequencies indicated, switchers were more common in fluctuating environments, whereas in stable environments, genetic solutions were more prevalent (fig. 5*A*). To further confirm that the ability to switch between the two phenotypes indeed depended on the epigenetic machinery, we additionally replica plated the 5-FOA-resistant clones onto media containing nicotinamide (NAM), a known inhibitor of SIR-mediated gene silencing (Bitterman et al. 2002). In the environments with periodicity corresponding to the switching rate (4-day fluctuation period), resistance to the drug was abrogated upon exposure to NAM indicating that adaptation depended on the epigenetic machinery in these clones. On the other hand, drug resistance of the evolved clones from the stable environment was unaffected by the presence of NAM, providing further evidence of the genetic basis of adaptation to these environmental conditions (fig. 5*B*). Surprisingly, in fluctuating environment with periodicity higher than the switching rate (1-day fluctuation period), we observed clones that still showed the ability to switch between the two-gene expression states, but in which resistance to 5-FOA was not altered by the addition of NAM. This indicates the existence of alternative genetic solutions that maintained the switching ability in these populations without altering the uracil biosynthesis pathway directly.

### The Effect of the Epigenetic Switching System Depends on its Rate of Change

Deletion of SIR3 can potentially have effects on different phenotypic traits. Null mutants of SIR3 are known to have impairment in mating, reflected both in the inability to respond to alpha factor (Chasse et al. 2006) and alteration in the meiotic division (Trelles-Sticken et al. 2003). However, we do not expect these effects to play a significant role in our experimental setup, because our study is using haploid yeast strains that cannot undergo sexual reproduction. Additionally, the absence of SIR3 is known to cause an increase in mutation rate in the subtelomeric region (Stajic et al. 2019), probably due to increased recombination rate. SIR3 gene disruption was associated with decreased viability under starvation conditions (Guidi et al. 2015) and abbreviated life span (Kaeberlein et al. 1999). Although studies using whole gene deletions have not detected significant changes in growth rate for Δsir3 mutants (Yoshikawa et al. 2011), these pleiotropic effects could potentially impact the results of the competition experiment. To ensure that the effect of epigenetic switching observed in the fluctuating environments was solely due to the intrinsic characteristic of the epigenetic system of gene expression control and not due to possible deleterious phenotypic effects of the *SIR3* knockout mutation, we performed an additional evolution experiment. We competed a yeast strain, referred to as slow epigenetic switcher, that has a lower rate of epigenetic switching (ON rate  ≈ 10^−2^, OFF rate  ≈ 10^−6^; Stajic et al. 2019), but otherwise has the same growth and mutation rate as the fast epigenetic switcher, with its corresponding Δsir3 mutant. The slow epigenetic switcher is preferentially in ON state of *URA3* expression and behaves very similarly to the non-switcher strain. Similar to the procedure in the first experiment, we preselected the cultures of the two strains in media lacking uracil and then exposed the cocultures to the three fluctuating environments (fig. 1).

**Fig. 6.**
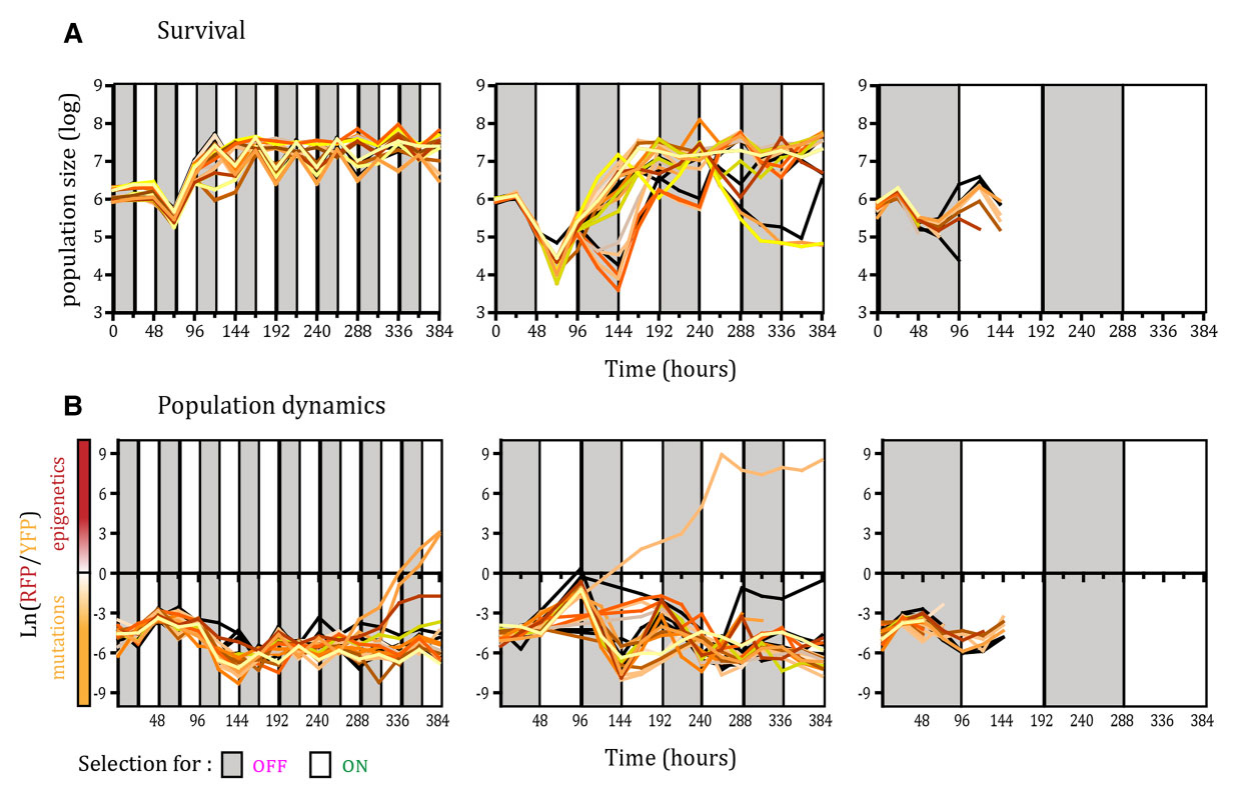
The dynamics of epigenetic switchers depend on the rate of epigenetic switching. (*A*) Survival through the course of selection for a strain with a low rate of epigenetic switching in the three fluctuating environments with distinct periodicities, determined using FACS methodology. Each line represents number of cells in each replicate population (24 replicate populations for each environmental condition). Colored areas indicate the selection regime, gray corresponds to selection for inactive *URA3* and white for selection for the active form of the gene. (*B*) Dynamics of RFP/YFP ratios (with a low rate of epigenetic switching) in the fluctuating environments. The logarithm of RFP/YFP ratios for each of the replicate populations is shown, determined using FACS methodology. The color of the line for each population corresponds to the color of the lines in survival graphs. Colored areas indicate the selection regime as in panel A. Positive values indicate dominance of RFP strain, and negative dominance of YFP strain.

We observed that populations tended to go extinct as the environmental period increased (fig. 6; supplementary table S1, Supplementary Material online). Under 1-day fluctuating periods, all populations survived, likely because the time spent in one environment was not enough to eliminate the maladaptive phenotypic state before the environment changed. On the other hand, under 2-day fluctuations, we observed extinction in 2/24 replicate populations, and under 4-day fluctuations, all 24/24 replicate populations went extinct. This is a very different outcome than that observed in the cocultures with the fast epigenetic switcher, for which under 4-day fluctuations all populations survived (*P* < 0.00001, Fisher’s exact test). Thus, the rate of epigenetic switching played an important role for the adaptation to fluctuating environments, further confirming theoretical predictions (Lachmann and Jablonka 1996). If the epigenetic switching rate is low, the populations may go extinct because they cannot respond quickly enough to the strong selection pressure. Under fast epigenetic switching, a more phenotypically heterogeneous population can be rapidly established and cope with fast environmental changes. Furthermore, we observed that the populations which survived the experiment in rapidly fluctuating environmental conditions were dominated by non-switcher strains. Indeed, in 21/24 surviving replicate populations under 1-day fluctuations and 19/22 surviving replicate populations under 2-day fluctuations showed a frequency of non-switcher strains that was above 90%. This is in contrast to the experiment with the fast switcher, in which the majority of populations were ultimately dominated by the switcher strain (fig. 3).

## Discussion

**Fig. 7.**
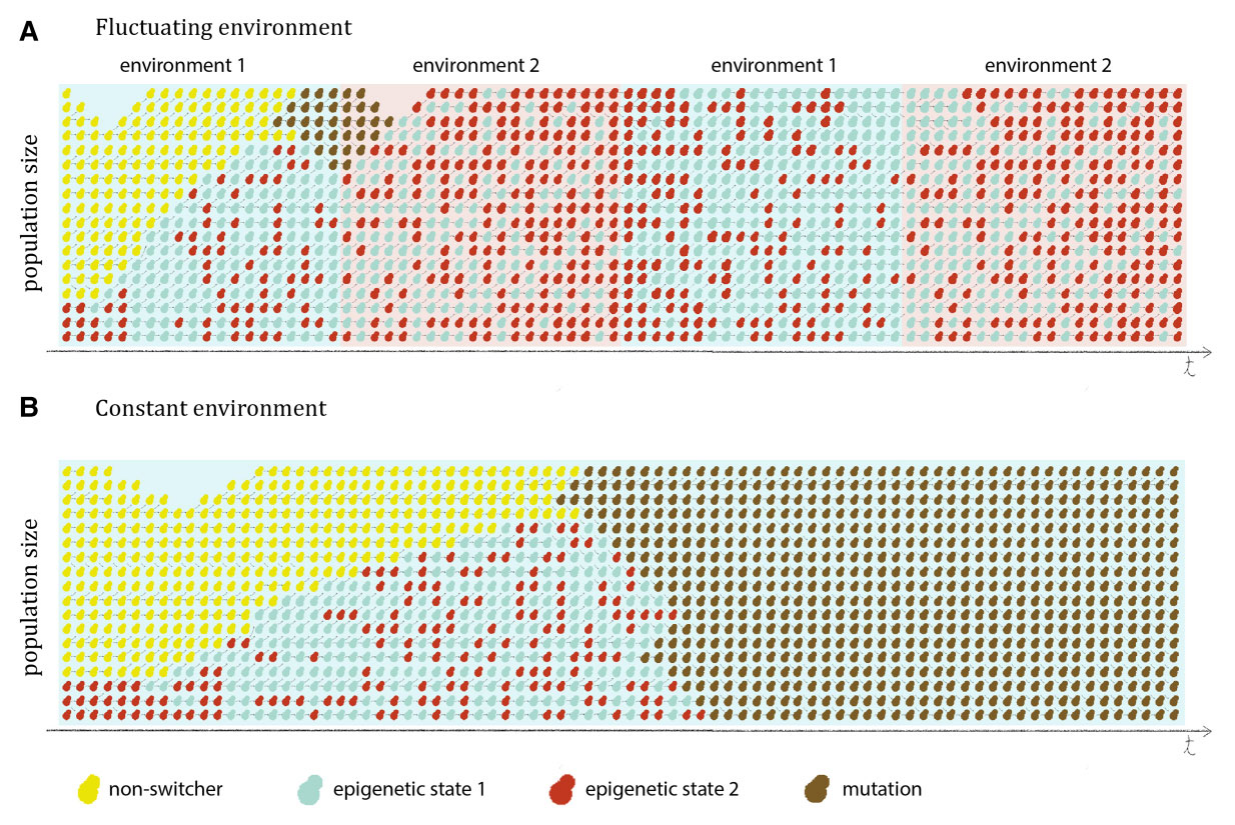
Schematic representation of the results. (*A*) In fluctuating environments where the conditions select for distinct gene expression state (blue and red background), mechanisms that enable stochastic switching between two phenotypes (red and blue cells) will be favored over a stable phenotypic determinant (yellow cells). Due to the slow rate of change, genetic mutations (brown cells) have little impact in a changing environment. (*B*) In a stable environment, epigenetic switching might provide an initial advantage to the survival of the population. However, once genetic mutations appear they would sweep to fixation.

Epigenetic control of gene expression can have profound effects on phenotypic variation (Jablonka and Raz 2009) and provide a bet-hedging survival strategy in variable environments. Epigenetic gene expression control is expected to be favored during adaptation to changing environments when the fluctuation period corresponds to the epigenetic switching rate, whereas mutations should be the predominant mechanism of adaptation in constant environments (fig. 7; Lachmann and Jablonka 1996).

Our results are in accordance with these theoretical predictions. In the constant environment selecting for *URA3* inactivation (5-FOA stable environment), we observed an initial rapid rise in frequency of the epigenetic switcher strain followed by a slow decrease in frequency and the establishment of an equilibrium frequency between switcher and non-switcher strains. This is in accordance with previously published results (Stajic et al. 2019) that showed epigenetically determined phenotypic states preceding the acquisition of adaptive mutations due to their higher rate of change. Indeed, phenotypic analysis showed that by the end of the experiment all of the evolved populations in this study adapted through the acquisition of mutations in the *URA3* biosynthesis pathway in both the switcher and non-switcher background. Surprisingly, cells capable of epigenetic control of *URA3* expression were still maintained in some populations under stable environmental conditions, even after 200 generations. In a constant environment selecting for *URA3* activation (i.e., an environment lacking uracil), we observed no noticeable change in frequency between the epigenetic switcher and non-switcher strains (fig. 3*B*). This is probably because the epigenetic switcher strain was preselected to be in the active ON state at the onset of the experiment. Additionally, whereas the two environments select for opposite phenotypic states, the selective pressure between them is different. 5-FOA is a drug that actively kills the cell, whereas the lack of uracil in the environment is not lethal. This difference between the environmental pressures is further corroborated by our results from the fluctuating environmental regimes, where we observe a decrease in the population size only in the environmental periods where 5-FOA is present (fig. 2*A*).

In fluctuating environments, all evolved populations maintained the ability to switch between the two-gene expression states (fig. 5). However, the molecular basis of this ability differed depending on the rate of the environmental change (fig. 5*B*). When the fluctuation period was faster than the epigenetic switching rate (1-day fluctuation), the evolved clones maintained the ability to switch. However, also in the presence of the inhibitor of the epigenetic machinery, some populations maintain the ability to switch, indicating that the mechanism of the switch is genetic in its nature. Mutations that confer resistance to 5-FOA but do not alter the uracil biosynthesis process, though rare, have been previously reported (Lang and Murray 2008). Meanwhile, in the environment in which the fluctuation period corresponded to the epigenetic switching rate (4-day fluctuation), the maintenance of gene expression states remained dependent on the epigenetic machinery. This empirical result seems to support previous theoretical studies (Lachmann and Jablonka 1996; Rajon and Charlat 2019).

Initially, we used the relative frequency of fast epigenetic switcher and nonswitcher clone (Δsir3 mutant) as a measure of the adaptive potential of epigenetic switching. However, these two strains might differ in other phenotypic characteristics apart from their ability to epigenetically silence the reporter gene. To ensure that the change in frequencies between the strains we observed in experiments is solely due to the different epigenetic silencing capacity and not to other possible phenotypic effects of *SIR3* gene deletion, we competed an epigenetic switcher strain with a lower frequency of switching (i.e., predominantly in ON state) with its corresponding Δsir3 mutant. Any detrimental phenotypic effect of the *SIR3* knockout is the same in the two competition set-ups, and the fast and the slow epigenetic switchers differ solely in their epigenetic switching rate (see Stajic et al. 2019). In competition with the slow epigenetic switcher, the frequency dynamics of Δsir3 mutant strain were markedly different from the competitions with the fast epigenetic switcher, indicating that the different patterns of adaptation we observed in the two competitions are indeed due to the difference in the epigenetic silencing.

Genetic and epigenetic systems of inheritance are highly inter-connected and inter-dependent, which makes differentiating the phenotypic effects of epigenetic changes from those of genetic mutations very difficult (Adrian-Kalchhauser et al. 2020). Using asexually reproducing, clonal, populations enabled us to precisely do this in a controlled system. However, to understand the generality of our observations, it is important to repeat such studies in more complex, sexually reproducing organisms. Partial or complete erasure of epigenetic marks which happens during gametogenesis probably adds another level of complexity.

Even though it still remains difficult to draw a causal link between adaptation and particular epigenetic marks in natural populations (Herrera and Bazaga 2011), ecological studies of epigenetic variation in nature have shown environment-dependent pattern of epigenetic marks (Richards et al. 2017; Heckwolf et al. 2020). However, the correlation between particular epigenetic gene expression patterns and the habitat seems to be species specific (Alonso et al. 2015; Niederhuth et al. 2016) and might be the result of environmentally induced epigenetic change (Seong et al. 2011; McCleary and Rine 2017; Richards et al. 2017).

Further indication of the adaptive advantage of epigenetic control of gene expression comes from budding yeast, where most of the genes in the subtelomeric region which were shown to be under epigenetic control are stress-related genes (Ellahi et al. 2015). These genes are nonessential under normal, stable conditions, but are responsible for quick physiological response to a sudden environmental insult. Having these genes under epigenetic control, which enables rapid transition from inactive to active form, might be beneficial in long-term.

In summary, our experimental setup offers a controllable and tractable system by which we can monitor the effects of heritable gene expression states and mutations during adaptation to different environments. Our observations show that an epigenetic system might provide opportunity for populations to adapt to rapidly changing conditions and prove important for survival under adverse environmental fluctuations (fig. 7). Additionally, epigenetic switching, as we have shown, could provide an additional layer for the maintenance of phenotypic and genotypic heterogeneity in the population.

## Materials and Methods

### Yeast Strains and Growth Conditions

All *S. cerevisiae* strains used in this study were derived from the S288c background. The fast epigenetic switcher strain (YIG1) was constructed by the integration of a NatMX6 (noursethricin resistance)-mCherry cassette into the original *URA3* locus (between 115,929 and 117,048 position on chromosome V) of the LJY186 strain (*MATα, trpΔ63*, *hisΔ200, ura3Δ::KanMX6, TEL-XIL::URA3 position 1373*), deleting the originally positioned Kanamycin-resistance cassette (KanMX6). The NatMX6-mCherry cassette was amplified from pDS3, constructed by insertion of an mCherry gene from pLJ760 into pAG25, containing a NatR cassette. The slow epigenetic switcher strain (YIG2) was constructed similarly, by the integration of an NatMX6-mCherry construct into the original *URA3* locus of the LJY185 strain (*MATα, trpΔ63*, *hisΔ200, ura3Δ::KanMX6, TEL-XIL::URA3 position 1623*). The YIG1 corresponding non-switcher strain (YIG3) was constructed by the integration of a NatMX6-mCitrine cassette into the original *URA3* locus of the LJY193 strain (*MATα, trpΔ63*, *hisΔ200, ura3Δ::KanMX6, sir3Δ::HYG, TEL-XIL::URA3 position 1373*). The NatMX6-mCherry cassette was amplified from pDS4, constructed by insertion of the mCitrine gene from pLJ761 into pAG25, containing a NatR cassette. The YIG2 corresponding non-switcher strain (YIG4) was constructed by the integration of a NatMX6-mCitrine cassette into the original *URA3* locus of the LJY192 strain (*MATα, trpΔ63*, *hisΔ200, ura3Δ::KanMX6, sir3Δ::HYG, TEL-XIL::URA3 position 1623*). The original plasmids and yeast strains used in the design and construction of strains in this study were a kind gift from Lars Jansen lab.

All strains were maintained either in rich medium (YPD; 1% Bacto yeast extract-BD [Fisher; #212720], 2% Peptone [Fisher; #BP1420-500], 2% Glucose [Merck; #1.08342.1000], either as liquid medium or supplemented with 2% Agar [Roth; #2266,4] for solid medium) or in complete synthetic dropout medium (CSM; 0.7% Yeast nitrogen base [Sigma; #Y0626], 0.1% complete synthetic medium [MP Biomedicals; #4560-222], 0.005% Tryptophan [Sigma; #T0254], 0.002% Histidine [Sigma; #H8000], 2% Glucose [Merck; #1.08342.1000] as liquid medium).

### Experimental Evolution

All strains were preselected to be in *URA3^+^* state by growing cells in liquid CSM lacking uracil for 16 h at 28 °C. Next, around 10^6^ cells in a 1:100 proportion of epigenetic switcher and non-switcher strain were diluted into 1 ml liquid CSM containing 5-FOA (CSM supplemented with 0.05% 5FOA [Apollo Scientific; #PC4054] and 0.001% Uracil [Sigma; #U0750]). Each day, 100 µl of the culture was placed into fresh media. Depending on the periodicity of environmental fluctuations (see fig. 1), medium was alternated between CSM containing 5-FOA and regular CSM. At each time point, 10 µl of the cultures was mixed into 190 µl of 1%PBS solution containing SPHERO fluorescent spheres (AccuCount 2.0 μm blank particles) that enabled accurate determination of the volumes. This mix was subsequently analyzed using flow cytometry to determine the number YFP- and RFP-labelled cells in each population. Exact number of cells in each population was obtained by the multiplication of detected events with proper dilution factor. If the total population size was less than 10^5^ cells for more than 4 time points, the population was considered extinct.

### Flow Cytometry

Flow Cytometry was performed in a BD LSR Fortessa™ SORP flow cytometer, using a 96-well-plate high-throughput sampler. The relative number of respective fluorescently labelled yeast cells in each replicate population was determined by the number of counts of detected fluorescent events and the appropriate dilution that was made in PBS solution. The instrument is equipped with 488 nm laser for scatter parameters and YFP detection and 561 nm laser for mCherry detection. Relative to the optical configuration, YFP and mCherry were measured using bandpass filters in the range of 540/30 and 630/75 nm, respectively. The analyzer was also equipped with a forward scatter detector in a photomultiplier tube to detect yeast. The results of the measurements were analyzed using Flowing Software version 2.5.1, developed by Perttu Terho, University of Turku. All flow cytometry experiments were performed at the Flow Cytometry Facility of Instituto Gulbenkian de Ciência, Oeiras, Portugal. The data from flow cytometry analyses are available in supplementary table S1, Supplementary Material online.

### Phenotypic Characterization of the Evolved Clones

We plated appropriate dilutions of each replicate population from the last time point on the rich media plates. The dilutions were made using the total cell numbers determined by flow cytometry so that around 100 cells were plated. The plates were incubated at 28 °C for 3 days. Subsequently, the colonies were counted and replica plated onto CSM plates containing 0.1% 5-FOA as well as onto regular CSM plates (without 5-FOA and without supplemented uracil). These plates were incubated at 28°C for 5 days, after which period the cells were counted. The frequency of epigenetic switchers was determined by dividing the number of colonies that grew on both CSM plates and the number of colonies on the rich media plates. Furthermore, the colonies from 5-FOA containing plates were replica plated on CSM plates that contained 0.1% 5-FOA and were additionally supplemented with 5 mM nicotinamide (Fisher; #1663C). These plates were incubated for additional 5 days at 28 °C, after which the colony number was scored. The frequency of clones with genetic changes was determined by the division of the colony number from plates with nicotinamide and colony number from the original rich media plates.

## Supplementary Material

Supplementary data are available at *Genome Biology and Evolution* online.

## Supplementary Material

evac065_Supplementary_Data

## Data Availability

The data from flow cytometry measurements are available in supplemental table S1, Supplementary Material online. All the strains used in the study are available upon request.
